# Self-Organizing Interval Type-2 Fuzzy Neural Network Compensation Control Based on Real-Time Data Information Entropy and Its Application in n-DOF Manipulator

**DOI:** 10.3390/e25050789

**Published:** 2023-05-12

**Authors:** Youbo Sun, Tao Zhao, Nian Liu

**Affiliations:** College of Electrical Engineering, Sichuan University, Chengdu 610065, China

**Keywords:** self-organizing interval type-2 fuzzy neural network error compensation, real-time data information entropy, intelligent control, n-DOF manipulators

## Abstract

In order to solve the high-precision motion control problem of the n-degree-of-freedom (n-DOF) manipulator driven by large amount of real-time data, a motion control algorithm based on self-organizing interval type-2 fuzzy neural network error compensation (SOT2-FNNEC) is proposed. The proposed control framework can effectively suppress various types of interference such as base jitter, signal interference, time delay, etc., during the movement of the manipulator. The fuzzy neural network structure and self-organization method are used to realize the online self-organization of fuzzy rules based on control data. The stability of the closed-loop control systems are proved by Lyapunov stability theory. Simulations show that the algorithm is superior to a self-organizing fuzzy error compensation network and conventional sliding mode variable structure control methods in control performance.

## 1. Introduction

The n-DOF manipulator, as the earliest application of robotic systems, still plays an important role in various fields such as industry, the military, medicine, aerospace, and so on. In the context of the future Industry 4.0 era, we have higher and higher requirements for the control accuracy of n-DOF manipulators [1,2]. The mechanical and electrical systems of the manipulator have strong uncertainty in practical applications (including non-modeled uncertainty and modeling parameter uncertainty [3]), strong coupling, large influence of control signal time delay error [4], large influence of control signal error [5,6], etc. These problems are the difficulties of current control research [7,8].

In response to the above problems, many scholars have conducted a lot of valuable research work. As a reference, a manipulator error compensation method based on deep belief network and error similarity was proposed by [9]. This method requires data reprocessing and uses particle swarm optimization (PSO) to optimize the network structure and parameters, which cannot theoretically guarantee the stability of the control process. An adaptive fuzzy trajectory tracking method was proposed by [10]. When this method is applied, the steady-state error of the step response is good, but the response speed is too slow, and there is a large time delay error when the sinusoidal input response is used. Otherwise, a new type of fuzzy control algorithm was proposed by [11]. In this method, a large peak error occurs when the manipulator moves to the point of commutation, and the jitter is obvious during the rotation process. The overall error is about 0.06 degrees, and the control effect is not very ideal. An adaptive fuzzy control method with asymptotic tracking performance was proposed by [12]. The step response time of this method is about 4 s, which cannot meet the control effect in practical applications. A control method based on the combination of dual encoders was proposed by [13]. Instead of compensating the actuator, it actually reduces the error generated by the encoder. This approach actually improves the parameter accuracy of the object model. A predictive control method based on feedback linearization to compensate the time delay error in the control process of the manipulator system was proposed by [14]. This method can restore the stability of the control system when the time delay of the control signal reaches about 1 s, but the control accuracy is not improved. In addition, during the control process, load, speed, motion direction, and ambient temperature all have an impact on the control accuracy. Using deep learning to achieve high-precision compensation, the time complexity is too high and the robustness is poor.

The sliding-mode control method has advantages in electromechanical robot control due to its own characteristics. The most important advantage is that sliding-mode control itself has a certain robustness [15], so the controller part of the multi-degree-of-freedom manipulator control system is suitable to use the method based on sliding-mode variable structure control plus error compensation. In recent years, sliding-mode control has been increasingly used as the core control method of various complex control systems [16,17]. The main reason is that sliding-mode control has advantages in stability proof, which facilitates stability analysis after adding intelligent control algorithms to it.

In the aspect of manipulator error compensation, the fuzzy control method is a suitable method [18,19]. The main advantage of a fuzzy system is that it can express the experience and knowledge of human understanding by mathematical formulas [20,21,22]. However, with the increasing requirements of control precision, the traditional simple fuzzy control system based on expert experience has been unable to meet the needs [23]. The real-time self-organizing fuzzy error compensation method based on control data can break through the shackles of expert experience to a large extent, make full use of various data in the control process, realize self-organization and self-adaptation of fuzzy rules, and make the system grow into the most suitable rules-governing objects [24,25]. However, the existing self-organizing control algorithms based on real-time data drive still fail to effectively solve the problems of huge amounts of information, increasing entropy, and high computing cost in the control process.

Through the above content, we can easily find out that some problems in trajectory tracking of the n-DOF manipulator systems that need to be addressed are summarized as follows.

(1) Most of the previous studies are aimed at suppressing or compensating for a certain type of interference [26,27,28,29]. However, as a multi-coupling complex system with fast state change, the manipulator system has multiple types of disturbances in the movement process, and the information entropy of real-time data in the control process is high, and most of the disturbances are time-varying random disturbances [30].
(2) While pursuing high precision, previous studies usually inevitably increase the complexity of the control algorithm and increase the time complexity of the control system calculation [31,32,33], thus making the response time to significantly longer. In the n-DOF manipulator control system, the state response speed is very fast and the controller response speed is very high. It is difficult for the existing research to be competent in this kind of control work.
(3) In the existing research of the fuzzy self-organizing control framework, the initial rules and structure of fuzzy neural network have a great influence on the control effect [34,35]. The fuzzy neural network usually is self-organized in the control process after setting one or several good initial rules. However, in complex object control, it is usually impossible to set a suitable initial rule in advance, and the network needs to learn by itself from the first rule.

Regarding the aforementioned problems, a new self-organizing type-2 fuzzy feedback neural network compensation control framework is proposed to handle the trajectory tracking problem of mechanical n-DOF manipulator system with many types of uncertain disturbances. The main contributions of this paper are listed as follows:

(1) An error compensation algorithm for a n-DOF manipulator is proposed, which compensates various types of error perturbations in various mechanisms by directly comparing input and output errors, and reduces the computational complexity of data-driven fuzzy algorithms when the amount of real-time data and information entropy are large. At the same time, the stability of the closed-loop control systems are proved by Lyapunov stability theory, and the simulation also confirms its effect.
(2) A fuzzy network structure self-organization method and parameter self-adaptation method are proposed for the control object with high real-time requirements of electromechanical system. The time complexity and response time of this method can meet the requirements of real-time control.
(3) A fuzzy rule self-organizing increase and decrease index function is proposed. This function does not need the overall information of the data set (such as the overall variance, mean, etc., of the data set) nor does it need to store the previous data information. It only needs the current data information at each moment to guide the self-organizing network to achieve reasonable rule increase and decrease. At the same time, this paper explains the reason why the function is suitable for fuzzy self-organizing network from the theoretical structure level of the function.

The remainder of the paper is organized as follows. The first section introduces the research background and research status of the multi-DOF manipulator and its error compensation method. The second section mainly introduces the kinematic modeling and dynamics modeling of the control object of the manipulator used in this paper. The third section mainly introduces the controller design method of the manipulator, including sliding mode variable structure control and self-organizing interval type-2 fuzzy neural network design. The fourth section analyzes the stability of the control framework, which shows that the system is stable and convergent. The fifth section shows the operation results of the control system in the simulation environment. The last section summarizes the research presented in this paper and gives an outlook on future research directions.

To show the mentioned symbols clearly, their corresponding explanations are presented in Nomenclature Section.

## 2. Modeling of the Robotic Arm

### 2.1. Kinematic Modeling of the Manipulator

**Figure 1:**
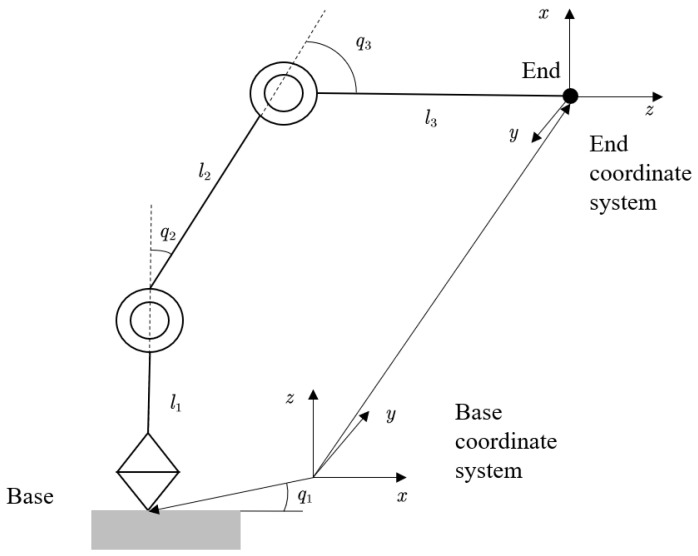
Object robotic arm kinematics modeling.

Kinematics studies the position, velocity, and acceleration of a rigid body in motion without considering the forces that produce the motion. In this paper, the D-H method is used to model the object’s n-DOF manipulator. The advantages of this method are that it involves few parameters, the model is consistent, and the coordinate system has 6 degrees of freedom. As shown in Figure 1, there are four parameters of the Denavit–Hartenberg (D-H) method [5,36]: *a*, α, *d*, and θ.

The kinematics problem of a multi-degree-of-freedom manipulator studies the mapping between the joint space and the end operation space of the manipulator. The forward kinematics problem refers to solving the motion parameters of the end of the manipulator given the input motion parameters of the joints. The inverse kinematics problem is the opposite, in which the motion parameters of the input joints are solved given the motion parameters of the end of the manipulator. In practical application, the user can only give the expected end motion trajectory and parameters of the manipulator, so the solution of inverse kinematics is more important. The kinematics model established in this paper is based on the right-handed coordinate system [37], as shown in Figure 1.

To sum up, the forward and inverse kinematic equations of the n-DOF manipulator designed in this paper are shown in (1) and (3), respectively.

(1) $$\left\{\begin{array}{c} x=\cos\left(q_{3}\right)\left(l_{1}\sin\left(q_{2}\right)+l_{2}\sin\left(q_{2}+q_{3}\right)\right)\\ y=\sin\left(q_{3}\right)\left(l_{1}\sin\left(q_{2}\right)+l_{2}\sin\left(q_{2}+q_{3}\right)\right)\\ z=l_{1}\cos\left(q_{2}\right)+l_{2}\cos\left(q_{2}+q_{3}\right) \end{array}\right.$$

(2) $$\left\{\begin{array}{c} a=l_{1}\sin\left(q_{2}\right)+l_{2}\sin\left(q_{2}+q_{3}\right)\\ b=l_{1}\cos\left(q_{2}\right)+l_{2}\cos\left(q_{2}+q_{3}\right)\\ k_{1}=l_{1}+l_{2}\cos\left(q_{3}\right)\\ k_{2}=l_{1}+l_{2}\sin\left(q_{3}\right)\\ \cos\left(q_{3}\right)=\frac{a^{2}+b^{2}-l_{1}^{2}-l_{2}^{2}}{2l_{1}l_{2}}\\ \sin\left(q_{3}\right)=\sqrt{1-\cos^{2}\left(q_{3}\right)}\\ \omega =\arctan(\frac{k_{2}}{k_{1}}) \end{array}\right.$$

(3) $$\left\{\begin{array}{c} q_{1}=\left\{\begin{array}{cc} \arctan(y/x), & len\neq 0\\ 0, & len=0 \end{array}\right.\\ q_{2}=\arctan\left(a/b\right)-\omega q_{3}=\arctan\\ \left(\sin\left(q_{3}\right)\cos\left(q_{3}\right)\right) \end{array}\right.$$

### 2.2. Dynamic Modeling of the Manipulator

The dynamic model of the n-DOF manipulator reflects the relationship between the motion of the manipulator and the force/torque it is subjected to. The three-degree-of-freedom manipulator discussed in this paper is a free-moving manipulator; that is, the movement of the manipulator is not affected by environment obstacles. The position of this manipulator can be uniquely determined by the joint variable *q*, and each *q* is independent of each other. Therefore, we choose $q=\left[q_{1},q_{2},\ldots ,q_{n}\right]$ as the generalized coordinate of the robot. In this paper, a three-DOF manipulator is modeled based on the second Lagrange equation.

For a three-DOF manipulator, as shown in Figure 1, the moment of inertia and inertia product in the three-dimensional direction are as follows [38]:

(4) $$\begin{array}{ccc} x_{c}=\frac{1}{m}\int \left(x\right)dm & y_{c}=\frac{1}{m}\int \left(y\right)dm & z_{c}=\frac{1}{m}\int \left(z\right)dm\\ I_{x}=\int \left(y^{2}+z^{2}\right)dm & I_{y}=\int \left(x^{2}+z^{2}\right)dm & I_{z}=\int \left(x^{2}+y^{2}\right)dm\\ I_{xy}=\int \left(xy\right)dm & I_{xz}=\int \left(xz\right)dm & I_{yz}=\int \left(yz\right)dm \end{array}$$

Because $\int_{link_{i}}dm=m_{i}$, a symmetric constant matrix can be obtained to describe the mass distribution of the rod:

(5) $$J_{i}=\left[\begin{array}{cccc} \frac{-I_{x}^{i}+I_{y}^{i}+I_{z}^{i}}{2} & I_{xy}^{i} & I_{xz}^{i} & m_{i}^{i}x_{ci}\\ I_{xy}^{i} & \frac{I_{x}^{i}-I_{y}^{i}+I_{z}^{i}}{2} & I_{yz}^{i} & m_{i}^{i}y_{ci}\\ I_{xz}^{i} & I_{yz}^{i} & \frac{I_{x}^{i}+I_{y}^{i}-I_{z}^{i}}{2} & m_{i}^{i}z_{ci}\\ m_{i}^{i}x_{ci} & m_{i}^{i}y_{ci} & m_{i}^{i}z_{ci} & m_{i} \end{array}\right]$$

Since there are identities for any pair of indicator functions,

(6) $$\sum_{i=1}^{n}\sum_{j=1}^{i}f\left(i,j\right)=\sum_{j=1}^{n}\sum_{i=j}^{n}f\left(i,j\right)$$

(7) $$\sum_{i=j}^{n}\sum_{k=1}^{i}f\left(i,k\right)=\sum_{k=1}^{n}\sum_{i=\max(j,k)}^{n}f\left(i,j\right)$$

The kinetic energy and potential energy of the entire robotic arm can be described as

(8) $$T≜\frac{1}{2}\sum_{j=1}^{n}\sum_{k=1}^{n}h_{jk}{\dot{q}}_{j}{\dot{q}}_{k}=\frac{1}{2}{\dot{q}}^{T}H\left(q\right)\dot{q}$$

(9) $$V=-\sum_{i=1}^{n}m_{i}{\bar{g}}^{T_{0}}A_{i}^{i}{\tilde{r}}_{\mathrm{ci}}$$

From (6) and (7), the Lagrange equation and its partial derivatives can be obtained as

(10) $$L=T-V=\sum_{k=1}^{n}h_{jk}{\ddot{q}}_{k}+\sum_{k=1}^{n}{\dot{h}}_{jk}{\dot{q}}_{k}$$

(11) $$\frac{\partial L}{\partial q_{j}}≜\frac{1}{2}{\dot{q}}^{T}\frac{\partial H}{\partial q_{j}}\dot{q}-g_{j}$$

It follows from the Lagrange equation that

(12) $$\frac{d}{dt}\frac{\partial L}{\partial \dot{q}}-\frac{\partial L}{\partial q}=Q$$

Available:

(13) $$\begin{array}{cc} \sum_{k=1}^{n}h_{jk}{\ddot{q}}_{k}+{\dot{q}}^{T}C_{j}\dot{q}+g_{j}=\tau_{j} & j=1,2,\ldots ,n \end{array}$$

Writing (13) in matrix form, the dynamic model of the ideal n-DOF manipulator can be mathematically described as

(14) $$H\left(q\right)\ddot{q}+C\left(q,\dot{q}\right)q+G\left(q\right)=\tau$$

In the actual environment, there are measurable and immeasurable external disturbances such as friction. Therefore, the dynamic mathematical model of the multi-degree-of-freedom manipulator is

(15) $$H\left(q\right)\ddot{q}+C\left(q,\dot{q}\right)q+G\left(q\right)+F\left(q,\dot{q},\ddot{q}\right)+\lambda =\tau$$

Among them, H(q) is the inertia matrix, $C(q,\dot{q})$ is the centripetal force matrix and the Coriolis force matrix, G(q) is the gravity matrix, $F(q,\dot{q},\ddot{q})$ is the uncertainty item composed of friction, and λ is the uncertainty item caused by parameter uncertainty and external interference. The dynamics model of the three-degree-of-freedom manipulator discussed in this paper is as follows [39]:

(16) $$\begin{array}{cc} \; & h_{11}=I_{1}+a_{1}\cos^{2}\left(q_{2}\right)+a_{2}^{2}\cos\left(q_{2}+q_{3}\right)\\ \; & +a_{3}\cos\left(q_{2}\right)\cos\left(q_{2}+q_{3}\right)\\ \; & h_{22}=I_{2}+a_{1}+a_{2}+2a_{3}\cos\left(q_{3}\right)\\ \; & h_{23}=h_{32}=a_{2}+a_{3}\cos\left(q_{3}\right)\\ \; & h_{33}=I_{3}+a_{2}\\ \; & h_{12}=h_{13}=h_{21}=h_{31}=0 \end{array}$$

(17) $$\begin{array}{cc} \; & c_{11}=-\frac{1}{2}a_{1}{\dot{q}}_{2}\sin\left(2q_{2}\right)-\frac{1}{2}a_{2}\left({\dot{1}}_{2}+{\dot{q}}_{3}\right)\sin\left(2q_{2}+2q_{3}\right)\\ \; & -a_{3}{\dot{q}}_{2}\sin\left(2q_{2}+q_{3}\right)-a_{3}{\dot{q}}_{3}\cos\left(2q_{2}\right)\sin\left(q_{2}+q_{3}\right)\\ \; & c_{12}=-\frac{1}{2}a_{1}{\dot{q}}_{1}\sin\left(2q_{2}\right)-\frac{1}{2}a_{2}{\dot{q}}_{1}\sin\left(2q_{2}+q_{3}\right)\\ \; & -a_{3}{\dot{q}}_{1}\sin\left(2q_{2}+q_{3}\right)\\ \; & c_{13}=-\frac{1}{2}a_{1}{\dot{q}}_{1}\sin\left(2q_{2}+q_{3}\right)\\ \; & -a_{3}{\dot{q}}_{1}\cos\left(2q_{2}\right)\sin\left(q_{2}+q_{3}\right)\\ \; & c_{22}=-a_{3}{\dot{q}}_{3}\sin\left(q_{3}\right)\\ \; & c_{23}=-a_{3}\left({\dot{q}}_{2}+{\dot{q}}_{3}\right)\sin\left(q_{3}\right)\\ \; & c_{32}=-a_{3}{\dot{q}}_{2}\sin\left(q_{3}\right)\\ \; & c_{21}=-c_{12}\\ \; & c_{31}=-c_{13}\\ \; & c_{33}=0 \end{array}$$

(18) $$\begin{array}{cc} \; & f_{1}=\lambda_{1}sgn\left({\dot{q}}_{1}\right)\\ \; & f_{2}=\lambda_{2}sgn\left({\dot{q}}_{2}\right)\\ \; & f_{3}=\lambda_{3}sgn\left({\dot{q}}_{3}\right) \end{array}$$

(19) $$\begin{array}{cc} \; & g_{1}=0\\ \; & g_{2}=b_{1}\cos\left(q_{2}\right)+b_{2}\cos\left(q_{2}+q_{3}\right)\\ \; & g_{3}=b_{2}\cos\left(q_{2}+q_{3}\right) \end{array}$$

The three-degree-of-freedom manipulator system shown in (15) has the following characteristics [40]:

(1) The inertia matrix H is a symmetric positive definite matrix and bounded, that is
  (20) $$0<\lambda_{m}\left(H\right)\leq ∥H∥\leq \lambda_{M}\left(H\right)$$
(2) $\dot{H}\left(q\right)-2C\left(q,\dot{q}\right)$ is an obliquely symmetric matrix, that is
  (21) $$\begin{array}{cc} {ı}_{T}\left(\dot{H}\left(q\right)-2C\left(q,\dot{q}\right)\right) & \forall \zeta \in R^{n} \end{array}$$

## 3. Design of Robotic Arm Controller

**Figure 2:**
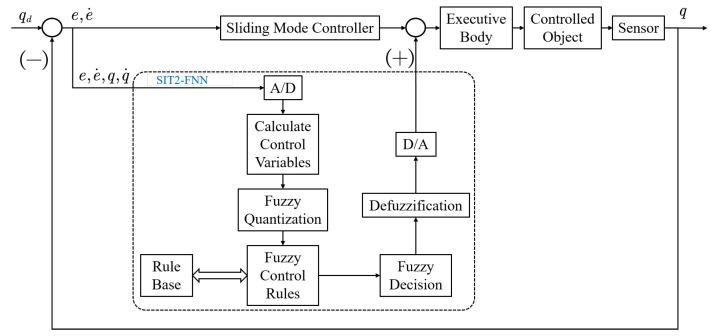
Overall structure block diagram of control system.

In this paper, aiming at the manipulator object to be controlled, the sliding mode control is used as the coarse tracking controller, T2-SOFNNEC is used as the fine tracking controller, and the total control signal is the output control signal of the two controllers. The block diagram of the control system is shown in Figure 2.

### 3.1. Design of Sliding Mode Controller

In this paper, the manipulator shown in Section 2 (15) is used as the control object to design the sliding mode controller. The goal of robot trajectory tracking control is to require the actual joint angular displacement vector $q={\left[\begin{array}{ccc} q_{1}, & q_{2}, & q_{3} \end{array}\right]}^{T}$ to track the desired joint angular displacement vector $q_{d}={\left[\begin{array}{ccc} q_{d1}, & q_{d2}, & q_{d3} \end{array}\right]}^{T}$ as well as possible.

The tracking error is defined as

(22) $$e=q_{d}-q$$

The sliding surface is designed as

(23) $$s=\Lambda e+\dot{e}$$

Among them, $s={\left[\begin{array}{ccc} s_{1}, & s_{2}, & s_{3} \end{array}\right]}^{T},\Lambda =\mathrm{diag}\left(r_{1},r_{2},r_{3}\right)$ is a positive diagonal matrix, and $\begin{array}{cc} r_{i}>0, & i=1,2,3 \end{array}$ is the slope of the sliding mode surface. Since the uncertain items in the model cannot be known, the object model is simplified as

(24) $$H\left(q\right)\ddot{q}+C\left(q,\dot{q}\right)q+G\left(q\right)+F\left(q,\dot{q},\ddot{q}\right)=\tau$$

The silding mode controller is designed as

(25) $$\tau_{\mathrm{eq}}=H{\ddot{q}}_{d}+H\Lambda \dot{e}+C\dot{q}+G+F+Cs+Ps$$

Among them, $P=diag(p_{1},p_{2},p_{3})$ is a positive diagonal matrix, $\begin{array}{cc} p_{i}>0, & i=1,2,3 \end{array}$

### 3.2. Design of Self-Organizing Interval Type-2 Fuzzy Error Compensation Controller (T2-SOFNNEC)

The type-2 fuzzy system is further fuzzified on the basis of the type-1 fuzzy system.The single membership function in Type-1 fuzzy systems is extended to the upper membership function and the lower membership function. Its fuzzy logic system can be described by the following “IF-THEN” statement:

(26) $$\begin{array}{cc} R^{\left(j\right)}:\mathrm{IF}\;x_{1}\;\mathrm{is}\;{\tilde{A}}_{n}^{i},\;\mathrm{THEN}\;y\;\mathrm{is}\;B^{j} & j=1,2,\ldots ,M \end{array}$$

where $R^{\left(j\right)}$ represents the *j*th rule; $\tilde{A}$ is the antecedent interval type-2 fuzzy set; ${\tilde{A}}_{n}^{i}$ represents the *i*th rule of the *j*th input; $B^{j}$ represents the consequent type-1 fuzzy set.

The most important difference between the type-2 fuzzy system and the type-1 fuzzy system is the membership function. The type-2 fuzzy is a three-dimensional surface, *x* is the input, *u* is the primary degree of membership, and $\mu_{A}\left(x,u\right)$ is the secondary degree of membership. In the interval type-2 fuzzy set $\mu_{A}\left(x,u\right)\equiv 1$, this greatly reduces the amount of computation.

**Figure 3:**
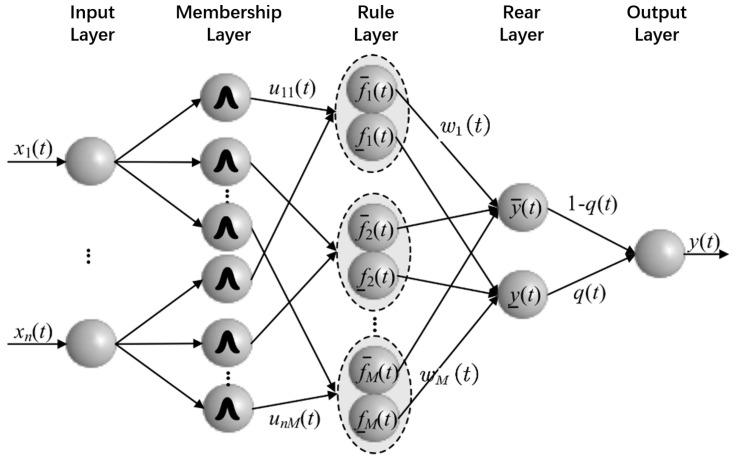
Interval type-2 fuzzy neural network structure diagram.

In order to make full use of the information related to the error in the control process, this paper uses the error, the error rate of change, the angle, and the angular velocity as the reference input of the error compensation algorithm, and the output is a compensation control signal. Obviously, the error compensation loop is a multi-input single-output structure. At the same time, the response ability of the proposed control algorithm should be ensure at the structural level. Therefore, this paper adopts the structure design compensation loop algorithm of IT2-FNN. The structure of the IT2-FNN is shown in Figure 3. The structure is mainly divided into five layers:

(1) Input layer: The input layer is the transmission of the reference input, and each neuron represents an input dimension.
(2) Membership layer: Each neuron in the membership layer represents the antecedent of a fuzzy rule, and each membership function takes the Gaussian function as the basis function, which can be expressed as
  (27) $$\left\{\begin{array}{cc} \; & {\bar{\mu }}_{ij}=e^{-\frac{1}{2}\frac{{\left(x_{ij}-c_{ij}\right)}^{2}}{{\left(\sigma_{ij}+\Delta \sigma \right)}^{2}}}\\ \; & {\underline{\mu }}_{ij}=e^{-\frac{1}{2}\frac{{\left(x_{ij}-c_{ij}\right)}^{2}}{{\left(\sigma_{ij}-\Delta \sigma \right)}^{2}}} \end{array}\right.$$
  Among them, $c_{ij}$ represents the center of the *j*th rule input in the ith dimension, $\sigma_{ij}$ represents the width of the *j*th rule input in the *i*th dimension, and Δσ represents the half interval width of the membership function.
(3) Rule layer: The value of each neuron in the rule layer is normalized by the corresponding membership degrees of all inputs, which can be expressed as
  (28) $$\left\{\begin{array}{cc} \; & {\bar{f}}_{j}=\prod_{j=1}^{M}\bar{\mu }ij\\ \; & {\underline{f}}_{j}=\prod_{j=1}^{M}\underline{\mu }ij \end{array}\right.$$
(4) Consequent layer: The output of the consequent layer is calculated by multiplying the rule value by the weight:
  (29) $$\left\{\begin{array}{cc} \; & \bar{y}=\sum_{j=1}^{M}\omega_{j}\times {\bar{f}}_{j}\\ \; & \underline{y}=\sum_{j=1}^{M}\omega_{j}\times {\underline{f}}_{j} \end{array}\right.$$
(5) Output layer: The function of the output layer is to downgrade the interval type-2 fuzzy system, and its output value is the product of the consequent value and the proportional coefficient:
  (30) $$y\left(t\right)=\bar{y}\left(t\right)\left(1-q\left(t\right)\right)+\underline{y}\left(t\right)q\left(t\right)$$

According to the above description, the biggest difference between the algorithm proposed in this paper and other type-2 fuzzy neural network structures is that although this paper adopts the interval type-2 fuzzy structure with a large amount of calculation, the depth and number of nodes of the fuzzy neural network are small, and there is no crossover between different rule antecedents. In fact, the algorithm proposed in this paper makes use of the stronger ability to deal with uncertain items of interval type-2 fuzzy sets and adopts a concise network structure so that the controller can achieve the best control effect with the fewest rules in the control process, thereby reducing the number of rules. A large number of crossover operations are used to reduce the time complexity of the control algorithm.

**Figure 4:**
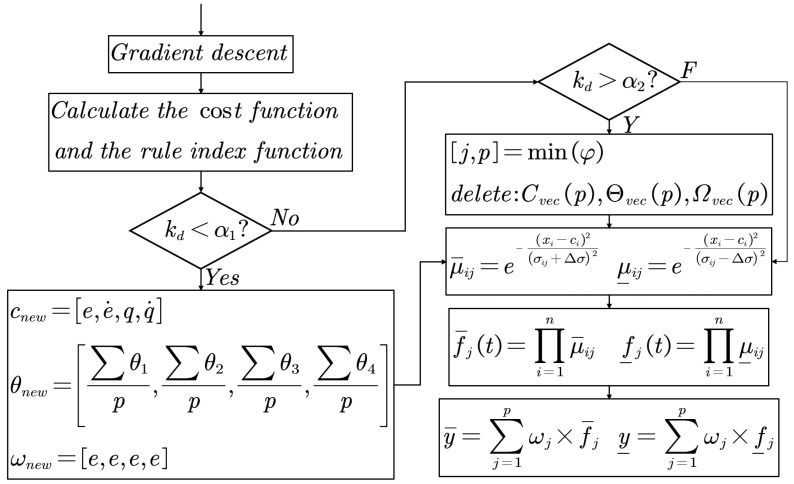
Interval type-2 fuzzy neuron network structure diagram.

In order to realize the real-time self-organization in the control process, a rule increase and decrease function is proposed in this paper, and a rule increase and decrease self-organization algorithm is designed based on this function. The self-organizing algorithm and its nested structure in the IT2-FEC algorithm are shown in Figure 4.

At the beginning of each control cycle, if there is no rule, a new rule is directly generated, and then the fuzzy network is calculated. If there is already a rule, we first update the network structure parameters according to the current input data and calculate the cost function and the rule increase or decrease index function. Then, according to the index function, it judges whether the rules need to be added or deleted. In order to ensure that the control algorithm always has a fast real-time response, each control cycle can perform rule addition or deletion at most once. After completing the self-organization of the rules, the control algorithm obtains the control signal through the fuzzy neural network calculation, and so on. The important components of the proposed control algorithm are described below.

(1) Estimation error

In order to guide the network structure and parameter learning, a network structure risk assessment model is proposed, which is defined as [41]

(31) $$\left\{\begin{array}{cc} \; & \rho_{1}=\frac{\sqrt{K\log(T_{g})}-2\log\left(K\right)}{\sqrt{T_{g}}}\\ \; & T_{g}=\frac{T}{t_{g}} \end{array}\right.$$

where *K* is the current total number of rules, *T* is the current moment, and $t_{g}$ is the current cycle number. The estimation error is an evaluation of the risk of the network structure.

(2) Experience error

The empirical error of the fuzzy network is defined as [41]

(32) $$\rho_{2}=\frac{1}{2}e^{T}e$$

Obviously, the empirical error index evaluates the compensation effect of the current network on the error on the one hand, and measures the adaptability of the network to new samples on the other hand.

(3) Rule increase and decrease function

In the self-organizing algorithm, when the controller increases the rules and when the rules are reduced is one of the most important steps and requires designing a reasonable and reliable rule increase and decrease function to help the controller determine whether to increase or reduce the rules. However, in the past, there was no paper to systematically design and evaluate the rules indicator function, and no paper explained the working mechanism of the rules to increase or decrease index functions.Therefore, a rule increase and decrease function is proposed in this paper. Otherwise, the working mechanism of the function during the control process is explained later.

Synthesizing the estimated error index and empirical error index of the network, the rule increase and decrease function is defined as

(33) $$k_{d}=\frac{\sqrt{\log(T_{g}/T_{g}K)}}{2}-\frac{2}{\sqrt{T_{g}}K}$$

**Figure 5:**
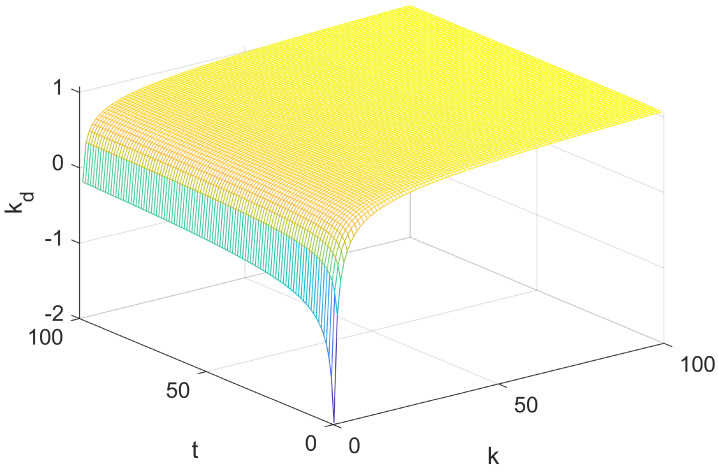
Schematic diagram of rule increase and decrease index function.

**Figure 6:**
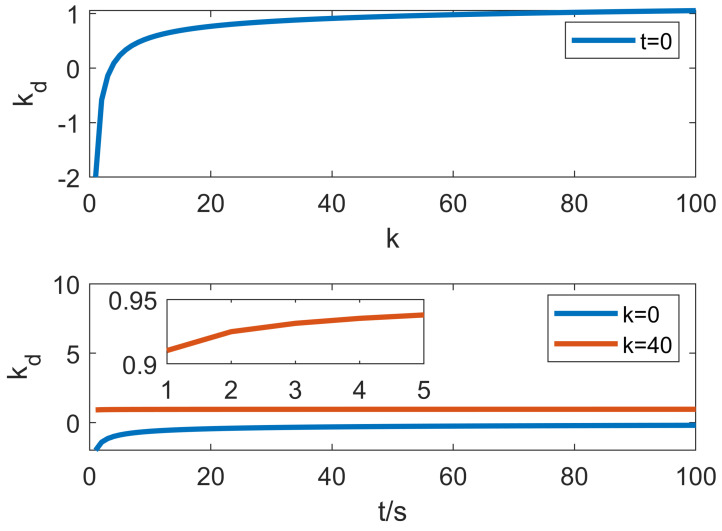
The trend of *k* and *t* in the exponential function.

The graph of this function is shown in Figure 5, from which we can see that the function value increases with the number of rules, while the partial derivative with time is different under different number of rules. It can be seen from Figure 6 that when the time is small, if the number of rules is large, the function value is too large, and the system adjusts by deleting rules; if the number of rules is small, the function value will be too small, and the system will adjust by adding rules. When the time is long, the function value tends to be stable, avoiding the system frequently adding and deleting rules and wasting computation.

(4) Rule growth stage

If $K_{d}<\alpha_{1}$, the network will add a new membership layer neuron to improve the generalization performance of the network. The parameters of the neurons newly added to the network are

(34) $$\begin{array}{cc} \; & c_{new}=x\left(t\right)\\ \; & \theta_{new}=\frac{\sum \theta }{M}\\ \; & \omega_{new}=e \end{array}$$

where $c_{new}$ is the center of the new neuron, x(t) is the current input value, $\theta_{new}$ is the width of the new neuron, and $\omega_{new}$ is the weight of the new neuron. When the network structure is adjusted, its parameters are updated as

(35) $$\begin{array}{cc} \; & C\left(t+1\right)=[C\left(t\right);c_{new}]\\ \; & \Theta \left(t+1\right)=[\Theta \left(t\right);\theta_{new}]\\ \; & \Omega \left(t+1\right)=[\Omega \left(t\right);\omega_{new}] \end{array}$$

where C(t),Θ(t),Ω(t) are the regular center matrix, width matrix and weight matrix of the network, respectively. It can be seen from (34) that every time a rule is added to the fuzzy set, it means that the data information of the new rule precedents appears in the discussion domain area with low probability. In this case, the fuzzy system covers the situation of the system information entropy being larger, which is the self-organization principle of the proposed algorithm.

(5) Rule deletion stage

In order to avoid the rule explosion caused by the continuous entropy increase of the system, the rule deletion stage plays an important role in the self-organizing algorithm. If $k_{d}>\alpha 2$, the redundant neuron with the smallest activation function in the network will be deleted. The redundant parameter membership neuron will be set to:

(36) $$\begin{array}{ccc} c_{j}\left(t+1\right)=0 & \theta_{j}\left(t+1\right)=0 & \omega_{j}\left(t+1\right)=0 \end{array}$$

(6) Parameter optimization stage

In order to improve the generalization performance of the fuzzy neural network, this paper uses the gradient descent method to optimize the structural parameters in each cycle. The optimization method is as follows:

(37) $$\begin{array}{cc} \; & C(t+1)=C(t)+\Delta C(t)\\ \; & \Theta (t+1)=\Theta (t)+\Delta \Theta (t)\\ \; & \Omega (t+1)=\Omega (t)+\Delta \Omega (t) \end{array}$$

where

(38) $$\begin{array}{cc} \Delta c_{j}\left(t\right) & =\frac{\partial \rho_{1}}{\partial c_{j}\left(t\right)}\\ \; & =\frac{x\left(t\right)-c_{j}\left(t\right)}{\sigma_{j}^{2}}\sum_{\upsilon =1}^{m}\left(\rho_{j\upsilon }\varphi_{j}\left(1-\varphi_{j}\right)e_{\upsilon }\left(t\right)\right)\\ \; & \upsilon =1,2,\ldots ,m\\ \Delta \theta_{j}\left(t\right) & =\frac{\partial \rho_{1}}{\partial \theta_{j}\left(t\right)}\\ \; & =\frac{{\left(x\left(t\right)-c_{j}\left(t\right)\right)}^{2}}{\sigma_{j}^{3}}\sum_{\upsilon =1}^{m}\left(\omega_{j\upsilon }\varphi_{j}\left(1-\varphi_{j}\right)e_{\upsilon }\left(t\right)\right)\\ \Delta \omega_{j}\left(t\right) & =\frac{\partial \rho_{1}}{\partial \omega_{j}\left(t\right)}=\varphi e\left(t\right) \end{array}$$

In the past, because the designed control object is a slow -changing object, the real-time requirements of the control algorithm are not high. Many various T2-FNN were proposed with a complicated self-organizing algorithm, which causes the algorithm to sacrifice more fastness under the requirements of the pursuit of control accuracy [42]. In fact, this is also a difficult point in the current high-level self-organized fuzzy algorithm. However, The above-mentioned self-organizing algorithm proposed in this article only increases or deletes the rules in a cycle cycle, and there is only one parameter correction, which not only greatly improves the real-time response capacity of the control algorithm but also obtains the requirements in organizing learning. It is obvious that the proposed algorithm can meet the complicated systems such as fastness and control accuracy of the robotic arm movement control system.

## 4. Stability Analysis

### 4.1. Stability Analysis of Sliding-Mode Control Loop

For (25), choose the Lyapunov function as

(39) $$V=\frac{1}{2}s^{T}Hs$$

Then,

(40) $$\dot{V}=\frac{1}{2}s^{T}\dot{H}s+s^{T}H\dot{s}=\frac{1}{2}s^{T}\left(\dot{H}-2C\right)s+s^{T}Cs+s^{T}H\dot{s}$$

Substituting (21) into (40) gives

(41) $$\dot{V}=s^{T}Cs+s^{T}H\dot{s}$$

Bringing the above formula into (25), we can obtain

(42) $$\begin{array}{cc} \; & H{\ddot{q}}_{d}+H\Lambda \dot{e}+C\dot{q}+G+F+Cs+Ps\\ \; & =H\ddot{q}+C\dot{q}+G+F \end{array}$$

Then,

(43) $$\begin{array}{cc} H\ddot{e}+H\Lambda \dot{e} & =-Cs-Ps\\ \Rightarrow H\dot{s} & =-Cs-Ps \end{array}$$

Bringing (43) into (41) gives

(44) $$\begin{array}{cc} \dot{V} & =s^{T}Cs+s^{T}\left(-Cs-Ps\right)\\ \; & =-s^{T}Ps \end{array}$$

Since the matrix *P* is positive definite, it can be obtained from (44) that

(45) $$\dot{V}<0$$

It can be seen from (39) and (45) that the Lyapunov function *V* is positive definite and the $\dot{V}$ is negative define. According to Lyapunov’s global stability theorem, the sliding-mode control system designed in this paper is globally asymptotically stable at the equilibrium point.

### 4.2. Stability Analysis of Self-Organized Growth Stage

In the growth stage, a new fuzzy rule is generated which changes the structure of the self-organizing interval type-2 fuzzy error compensation network. According to (34), when the network adds a new rule, its prediction error is

(46) $$E_{M+1}\left(t\right)=\frac{1}{2}{\left(y_{d}\left(t\right)-y_{M+1}\left(t\right)\right)}^{2}$$

According to (27)–(30), the above formula can be extended as

(47) $$\begin{array}{cc} E_{M+1}\left(t\right) & =\frac{1}{2}(y_{d}\left(t\right)\\ \; & -(q\left(t\right)\frac{\sum_{j=1}^{M}{\underline{f}}_{j}\left(t\right)\omega_{j}\left(t\right)+{\underline{f}}_{M+1}\left(t\right)\omega_{M+1}\left(t\right)}{\sum_{j=1}^{M}{\underline{f}}_{j}\left(t\right)+{\underline{f}}_{M+1}\left(t\right)}\\ \; & +(1-q\left(t\right)\frac{\sum_{j=1}^{M}{\bar{f}}_{j}\left(t\right)\omega_{j}\left(t\right)+{\bar{f}}_{M+1}\left(t\right)\omega_{M+1}\left(t\right)}{\sum_{j=1}^{M}{\bar{f}}_{j}\left(t\right)+{\bar{f}}_{M+1}\left(t\right)}))) \end{array}$$

where ${\underline{f}}_{M+1}$ and ${\bar{f}}_{M+1}$ are both less than unity. $\sum_{j=1}^{M}{\underline{f}}_{j}\left(t\right)\gg {\underline{f}}_{M+1}$,$\sum_{j=1}^{M}{\bar{f}}_{j}\left(t\right)\gg {\bar{f}}_{M+1}$. So we obtain

(48) $$\begin{array}{cc} E_{M+1}\left(t\right) & \approx \frac{1}{2}(y_{d}\left(t\right)-y\left(t\right)-(q\left(t\right)\frac{\omega_{M+1}\left(t\right)}{\sum_{j=1}^{M}{\underline{f}}_{j}\left(t\right)}\\ \; & +\left(1+q\left(t\right)\frac{\omega_{M+1}\left(t\right)}{\sum_{j=1}^{M}{\bar{f}}_{j}\left(t\right)}\right){))}^{2}\\ \; & <\frac{1}{2}{\left(y_{d}\left(t\right)-y\left(t\right)\right)}^{2}=E_{M}\left(t\right) \end{array}$$

According to the above analysis, when a new fuzzy rule is added to the network, its convergence speed is accelerated.

### 4.3. Stability Analysis of Self-Organizing Cut-Out Stage

When the network removes a rule, the prediction error of the network is

(49) $$E_{M-1}\left(t\right)=\frac{1}{2}{\left(y_{d}\left(t\right)-y_{M-1}\left(t\right)\right)}^{2}$$

According to (36), the above formula can be written as

(50) $$\begin{array}{cc} E_{M-1}\left(t\right) & =\frac{1}{2}(y_{d}\left(t\right)-(q\left(t\right)\frac{\sum_{j=1}^{M}{\underline{f}}_{j}\left(t\right)\omega_{j}\left(t\right)}{\sum_{j=1}^{M}{\underline{f}}_{j}\left(t\right)}\\ \; & +\left(1-q\left(t\right)\right)\frac{\sum_{j=1}^{M}{\bar{f}}_{j}\left(t\right)\omega_{j}\left(t\right)}{\sum_{j=1}^{M}{\bar{f}}_{j}\left(t\right)})\\ \; & -rule_{M-1}\left(t\right))\\ \; & =\frac{1}{2}{\left(y_{d}\left(t\right)-y\left(t\right)\right)}^{2}\\ \; & =E_{M}\left(t\right) \end{array}$$

Therefore, the convergence rate of the network remains unchanged after pruning a fuzzy rule.

Based on the above analysis and discussion, the three-DOF manipulator trajectory tracking control algorithm based on self-organizing interval type-2 fuzzy error compensation proposed in this paper can ensure its stability. The self-organizing link can ensure the convergence and even speed up the convergence in the parameter update stage, the rule addition stage and the rule deletion stage.

## 5. Simulation

In this paper, the control simulation is performed on the MATLAB2021 platform. The parameters of the target manipulator are shown in Table 1. The manipulator is controlled by SMC, T1-SOFNNEC [41] and T2-SOFNNEC.

### 5.1. Simulation with Base Jitter

**Figure 7:**
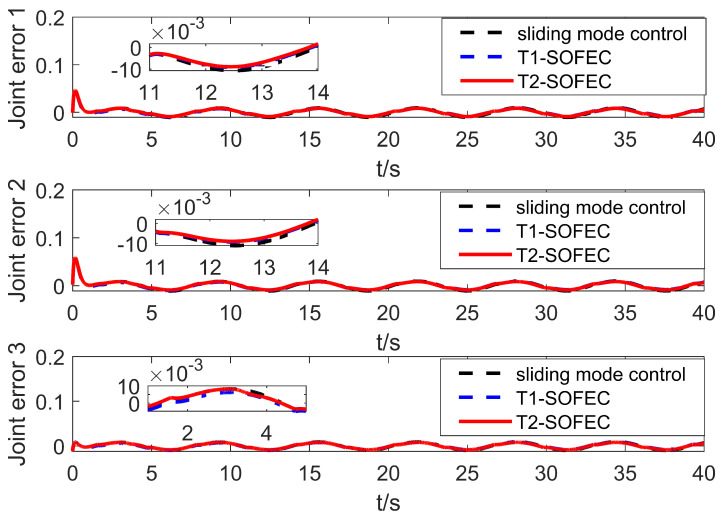
Error change curve with base jitter.

First of all, the effectiveness of the proposed control algorithm is verified in the case of only simulated base shaking. In the simulation, the pedestal jitter is expressed in the form of modeling uncertainty; that is, a time-varying random matrix of [0, 10] is added to the plant dynamics model. The control effects of SMC, T1-SOFNNEC and T2-SOFNNEC under the same conditions are compared as shown in Figure 7.

It can be seen from Figure 7 that all three control methods can realize the control of the manipulator. However, compared with the sliding-mode control, the self-organized fuzzy compensation control has a smaller error and higher control accuracy when the rotation direction of the manipulator joint changes. In addition, it can be seen from the figure that the self-organization method has a faster learning speed, and the control effect during the self-organization is almost the same as that of the sliding mode control.

### 5.2. Simulation with Base Jitter and Signal Interference

**Figure 8:**
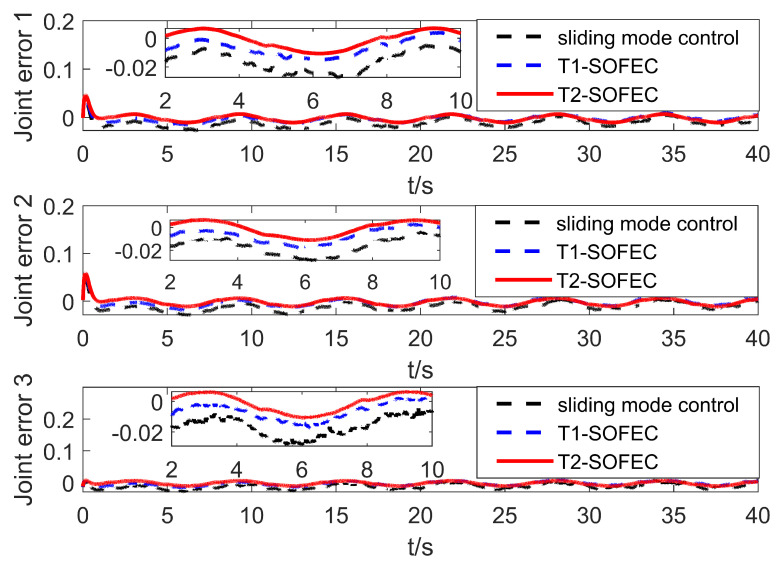
Error change curve with base jitter and signal interference.

In this part of the simulation, signal interference is added to the base jitter. In the simulation process, the manifestation of the signal interference is that the time-varying random interference signal of [0, 10] is added on the basis of the output control signal of the controller. The simulation results are shown in Figure 8.

It can be seen from Figure 8 that under the action of the self-organized fuzzy error compensation algorithm, the error fluctuation range of the three joints of the manipulator is significantly reduced, and the control accuracy is significantly improved. At the same time, the robustness of the self-organizing fuzzy error compensation algorithm is also improved; that is, the error jitter in Figure 8 is significantly reduced.

The RMSE of the three control framework in the presence of both jitter and control signal interference are shown in Table 2. It can be seen that the proposed T2-SOFNNEC has outstanding performance. This is due to the lifelong learning ability of the self-organizing method, which can adjust the controller structure according to the actual situation during the control process. At the same time, the interval type-2 fuzzy system itself has a strong ability to deal with uncertain terms, which has an advantage when multiple types of disturbances exist at the same time.

**Figure 9:**
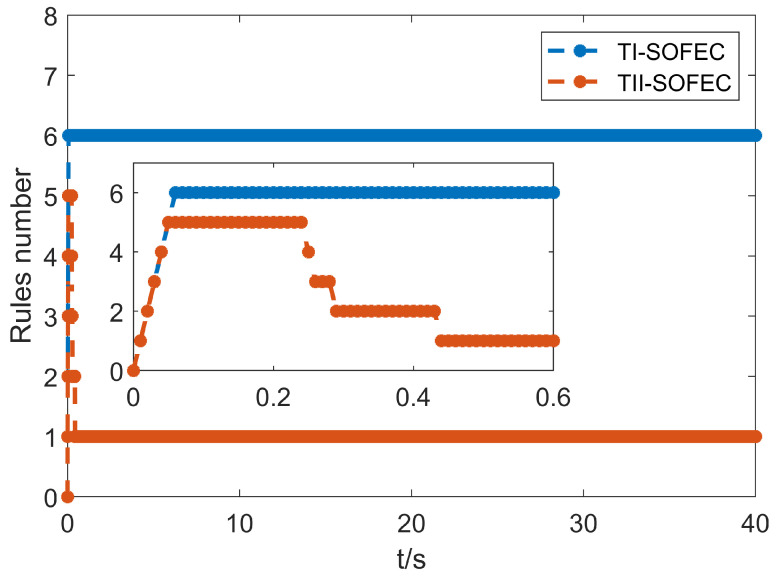
The rule number of two self-organizing fuzzy neural networks.

**Figure 10:**
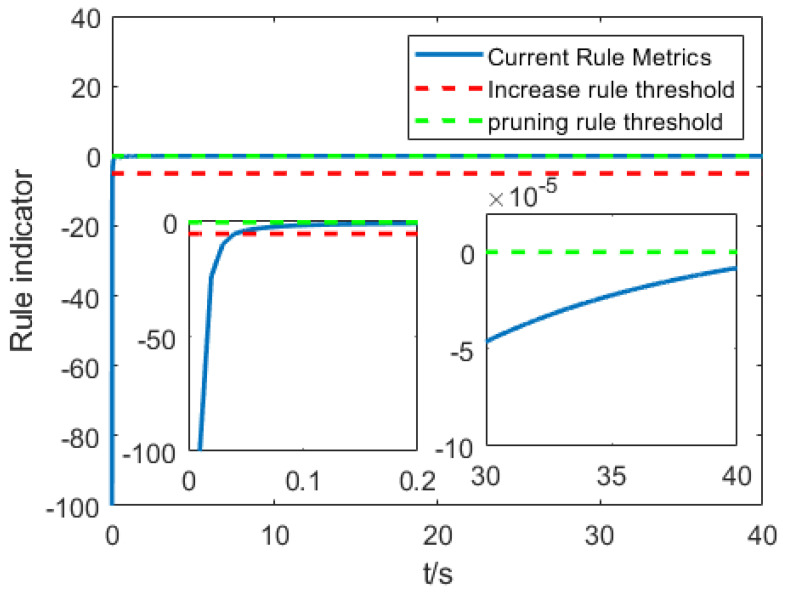
The index function of self-organizing type-2 fuzzy neural network.

The change in the rule number is shown in Figure 9. It can be seen that both algorithms are in the stage of adding rules at the beginning of the control, but the interval-type two algorithm gradually adjusts to only need one rule as the control time increases. However, the type-1 algorithm needs to be increased to six rules to compensate. In addition, the change of the rule index function in the control process of the proposed T2-SOFEC is shown in Figure 10, from which it can be seen that the proposed T2-SOFEC can regularly increase or decrease the index function during the control period between the thresholds. Therefore, the self-organizing interval type-2 fuzzy error compensation algorithm can achieve almost the same compensation effect with fewer rules than the self-organizing type-1 fuzzy error compensation algorithm, which makes it significantly reduce the time complexity in the control process, enhance the computational efficiency of the controller, and speed up the response time.

From this part of the simulation, it can be seen that the self-organizing fuzzy compensation algorithm has greater advantages than the previous algorithm and can compensate for errors to achieve better control effects in the presence of multiple disturbances. At the same time, under the same interference conditions, T2-SOFNNEC to T1-SOFNNEC uses fewer rules, which further reduces the storage and computation of the algorithm.

### 5.3. Simulation with Base Jitter, Signal Interference, and Time Delay

**Figure 11:**
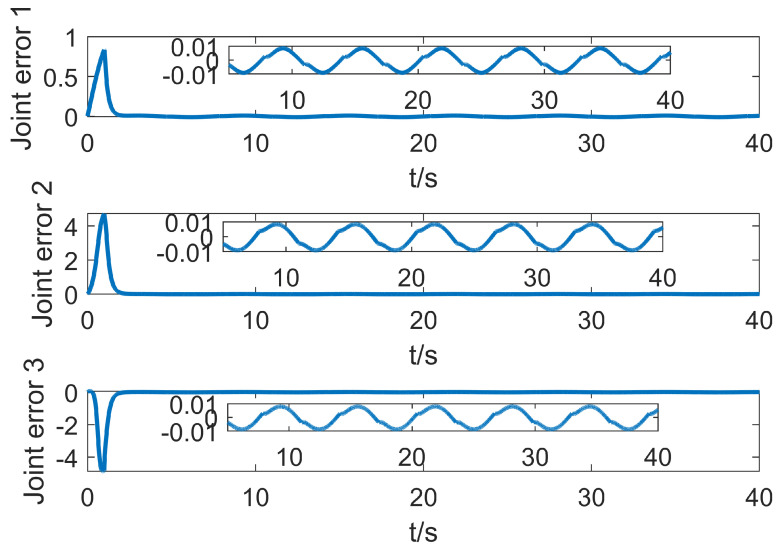
Error change curve with base jitter, signal interference and time delay.

Based on the simulation in the previous part, this part adds the time delay interference of the control signal to simulate the delay of the control signal communication in the actual control process. This situation generally exists in the control process of bus communication. In the simulation, the control delay shows that there is no control signal within one second when the control starts, and the control object receives the control signal output by the controller only after one second. When the system control signal delay is one second, the motion system error of the manipulator is shown in Figure 11.

It can be seen from Figure 11 that the system is in the uncontrollable divergence phase in the previous second. After one second, the T2-SOFNNEC can quickly stabilize the system and restore to a stable and controllable state. In the presence of time-delay error, the interval type-2 fuzzy system can make use of its more powerful advantages in dealing with uncertainty and make up for the limitation of insufficient number of rules. From another point of view, interval type-2 fuzzy error compensation trades less rules for its stronger advantage in dealing with the structure of uncertain terms.

### 5.4. Coppeliasim-MATLAB Co-Simulation

**Figure 12:**
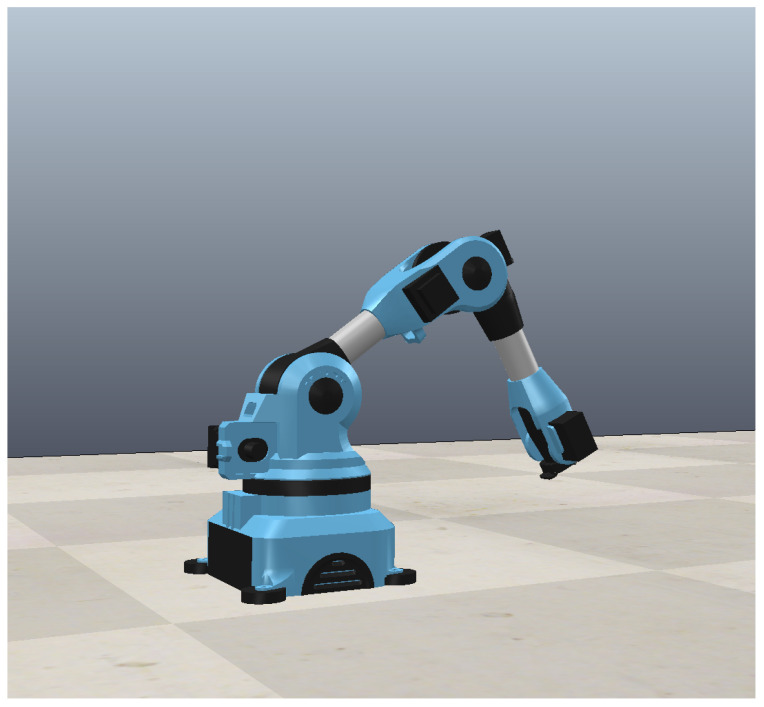
MATLAB-Coppeliasim co-simulation platform.

In order to further verify the feasibility of the method, a dynamic simulation environment such as Figure 12 is established on the Coppeliasim platform, and the control method proposed in this paper is verified by the MATLAB-Coppeliasim co-simulation method. Coppeliasim is a multi-platform robot simulation software that covers robot modeling, programming, and simulation functions. It has the characteristics of close to the real real control environment and supports multiple programming languages.

**Figure 13:**
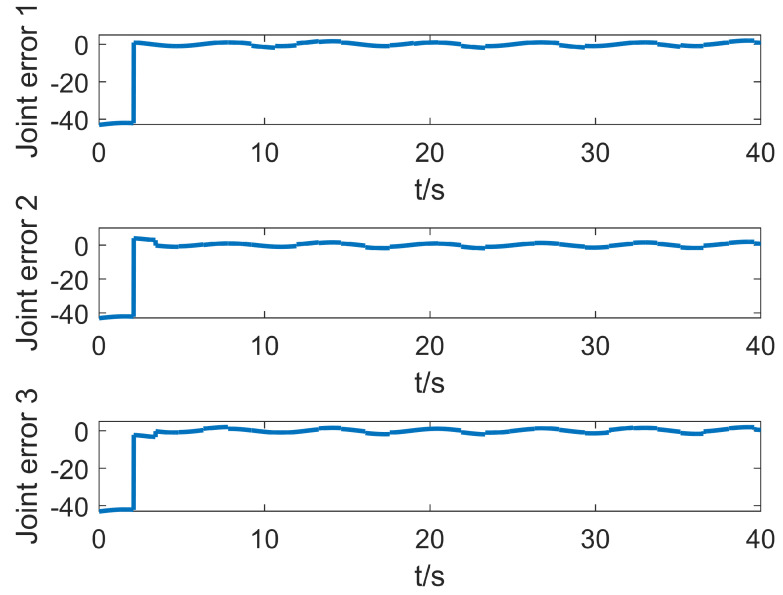
Co-simulation tracking error curve.

**Figure 14:**
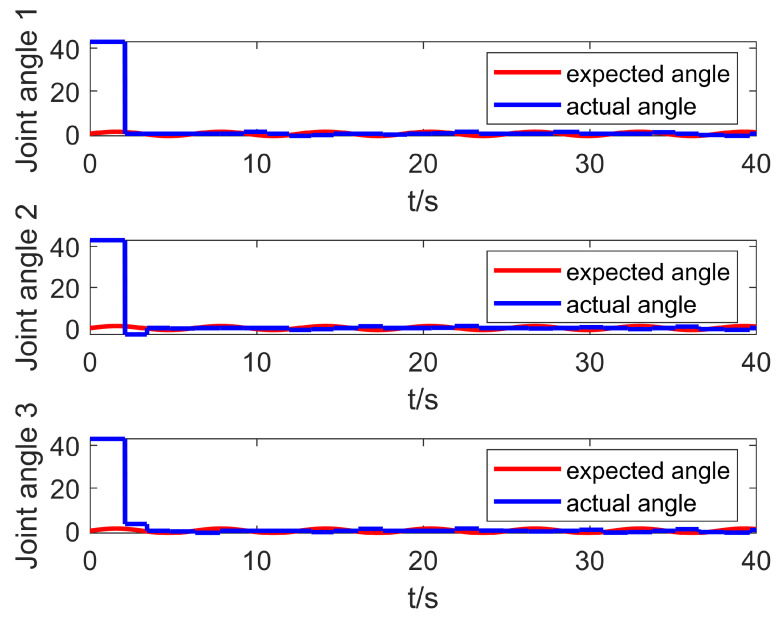
Co-simulation tracking effect curve.

The environment for co-simulation is the same as in the previous section, and the co-simulation results are shown in Figure 13 and Figure 14. It can be seen from Figure 14 that the initial state of the robotic arm is not at the origin; that is, the initial angle of the three joints is not zero, which is very consistent with the actual control process. In the first 2 s, the robotic arm did not receive a signal from the control system, and the robotic arm was in a disabled state at this time. At a certain moment, the robotic arm receives the time-delayed control signal sent by the controller, and at this time the robotic arm eliminates the error and suppresses the interference. It can be seen from Figure 13 that the control algorithm can eliminate the error near zero in a short time. It can also be seen from Figure 14 that the control method has a practical effect in the co-simulation and has a good control effect. Last but not least, the longest response time of the simulation control signal is 2 ms, which can fully meet the needs of real-time control.

## 6. Conclusions

The difficulty of trajectory tracking control of n-DOF manipulators under strong interference lies in how to achieve stable and high-precision control of the system under the condition of various types of interference, various interference, and large amounts of real-time information data. The proposed T2-SOFNNEC in this paper can better compensate for various disturbances in the system and their resulting effects under the condition that the structure is more concise, the calculation cost of self-organization and parameter tuning is lower, and the rules used are fewer so that it satisfies the effect of both real-time control and accuracy. Meanwhile, this paper also proposes a more interpretable self-organizing rule increase and decrease index function. This function does not need to store the previous data information or the overall information of the data set and can realize the self-organization of the number of rules under the condition of low storage cost and computational cost. The proposed algorithm in this paper uses the fuzzy neural network structure to ensure the stability of the control system and uses the structure self-organization and parameter adaptive methods to ensure high-precision control. In summary, the proposed algorithm based on T2-SOFNNEC is suitable for high-precision motion control of n-DOF manipulators with strong interference in practical situations.

## Figures and Tables

**Table 1:** Simulation robot-arm parameter table.

DOF	Link Mass (kg)	Link Length//(m)	Link Centroid//(m)
1	34	0	0
2	30	1	0.6
3	26	1	0.5
**DOF**	**Link’s Moment** **of Inertia** **about Its Axis** **of Rotation** **(kg·m${}^{2}$)**	**Coulomb** **Friction** **Coefficient**	**Gravitational** **Acceleration** **(m/s${}^{2}$)**
1	3.62	5	9.8
2	2.35	5	9.8
3	1.95	5	9.8

**Table 2:** RMSE of the three control frameworks.

Joint	SMC	T1-SOFNNEC	T2-SOFNNEC
1	0.026075	0.021213	**0.004734**
2	0.029306	0.024071	**0.004022**
3	0.024777	0.020316	**0.003727**

## Data Availability

The research data supporting this publication are provided within this paper.

## Nomenclature

$c_{ij}$	The ijth term of the centrifugal force matrix
*C*	Centrifugal force matrix
$c_{j}$	The center of the *j*th rule
*e*	Control-error signal
${\bar{f}}_{j}$	The upper output of the rule layer
${\underline{f}}_{j}$	The lower output of the consequent layer
$f_{i}$	Force of friction
*F*	Static and dynamic friction matrix
$g_{i}$	The *i*th gravitational vector
*G*	Gravitational vector matrix
$h_{ij}$	The ijth term of the inertial matrix
*H*	Inertial matrix
$k_{d}$	The index function
$l_{i}$	The length of the *i*th arm
$q_{d}$	Expected angle
$q_{i}$	The actual angle of the *i*th joint
$\bar{y}$	The upper output of the consequent layer
$\underline{y}$	The lower output of the consequent layer
*y*	The output of the FNN
α	The parameter of rule adaptive
$\theta_{j}$	The width of the *j*th rule
λ	The uncertain disturbance term caused by modeling error and external interference
${\bar{\mu }}_{ij}$	The value of Upper membership function
${\underline{\mu }}_{ij}$	The value of Lower membership function
$\rho_{1}$	Estimation error
$\rho_{2}$	Experience error
τ	Control moment
